# Single nucleotide polymorphisms in *LCAT* may contribute to dyslipidaemia in HIV-infected individuals on HAART in a Ghanaian population

**DOI:** 10.1038/s41598-020-76113-2

**Published:** 2020-11-10

**Authors:** Simon Bannison Bani, Kwabena Owusu Danquah, Christian Obirikorang, William K. B. A. Owiredu, Lawrence Quaye, Edmund Muonir Der, Emmanuel Acheampong, Yussif Adams, Peter Paul M. Dapare, Moses Banyeh, Enoch Odame Anto, Samuel Asamoah Sakyi

**Affiliations:** 1 Department of Biomedical Laboratory Sciences, School of Allied Health Sciences, University for Development Studies, Tamale, Ghana; 2 Department of Molecular Medicine, School of Medicine and Dentistry, College of Health Sciences, Kwame Nkrumah University of Science and Technology, Kumasi, Ghana; 3 Department of Medical Diagnostics, Faculty of Allied Health Sciences, College of Health Sciences, Kwame Nkrumah University of Science and Technology, Kumasi, Ghana; 4 Department of Pathology, School of Medicine and Health Sciences, University for Development Studies, Tamale, Ghana; 5 School of Medical and Health Sciences, Edith Cowan University, Joondalup, Australia

**Keywords:** HIV infections, DNA sequencing, Genetic predisposition to disease

## Abstract

Highly active antiretroviral therapy (HAART) is known to cause lipid abnormalities such as dyslipidaemia in HIV-infected individuals. Yet, dyslipidaemia may not independently occur as it may be worsened by single nucleotide polymorphisms (SNPs) in lecithin cholesterol acyltransferase (LCAT) and lipoprotein lipase (*LPL*). This case–control study was conducted in three-selected hospitals in the Northern part of Ghana. The study constituted a total of 118 HIV-infected participants aged 19–71 years, who had been on HAART for 6–24 months. Dyslipidaemia was defined based on the NCEP-ATP III criteria. HIV-infected individuals on HAART with dyslipidaemia were classified as cases while those without dyslipidaemia were grouped as controls. Lipid profile was measured using an automatic clinical chemistry analyzer and genomic DNA was extracted for PCR (GeneAmp PCR System 2700). Overall, the prevalence of dyslipidaemia was 39.0% (46/118). High levels of low-density lipoprotein cholesterol (LDL-C), total cholesterol (TC), and reduced levels of high-density lipoprotein cholesterol (HDL-C) were observed in all cases. A total of 256 selected PCR amplicons comprising 137 *LPL* (exons 3, 5 and 6) and 119 *LCAT* (exons 1, 4, and 6) were sequenced in 46 samples (Inqaba Biotech). Six (6) clinically significant SNPs were identified in exons 1 and 4 for *LCAT* whereas 25 non-clinically significant SNPs were identified for *LPL* in exons 5 and 6. At position 97 for *LCAT* exon 1, there was a deletion of the nucleotide, ‘A’ in 32.5% (13/40) of the sampled population while 67.5% (27/40) of the sample population retained the nucleotide, ‘A’ which was significantly associated with dyslipidaemic outcomes in the study population (p = 0.0004). A total of 25 SNPs were identified in exons 5 and 6 of *LPL*; 22 were substitutions, and 3 were insertions. However, none of the 25 SNPs identified in *LPL* exon 5 and 6 were statistically significant. SNPs in LCAT may independently contribute to dyslipidaemia among Ghanaian HIV-infected individuals on HAART, thus, allowing genetic and/or functional differential diagnosis of dyslipidaemia and creating an opportunity for potentially preventive options.

## Introduction

The incidence of human immunodeficiency virus (HIV) epidemic in Ghana is low with heterogeneously high prevalence in some key ethnic populations^1,2^. The prevalence rate of HIV among individuals within the ages of 15–49 years in Ghana has reduced from 2.3% in 2013 to 1.7% in 2018 with regional variation per the World Bank collection of development indicators^1–3^. The longevity of HIV patients has increased significantly in the past two decades due to the introduction of antiretroviral treatment (ART) as the gold standard treatment^4,5^.

In the management of HIV-infected individuals, highly active antiretroviral therapy (HAART) has been shown to significantly reduce HIV viral load, prevent the development of acquired immune deficiency syndrome (AIDS) and lower HIV related morbidity and mortality^6^. The current regimen is made up of two nucleotide reverse transcriptase inhibitors (NRTI) plus one non-nucleotide reverse transcriptase inhibitor (NNRTI) or two NRTI plus one protease inhibitor (PI)^7–9^. Despite the benefits derived from the use of HAART, there have been reports indicating that long-term usage of HAART induces significant adverse cardiometabolic and cardiovascular events such as dyslipidaemia, insulin resistance, central adiposity and lipodystrophy^10,11^. Among these events, dyslipidaemia has commonly been associated with prolonged use of HAART thus, increasing the risk of atherosclerosis and cardiovascular disease among HIV infected individuals^12,13^.

Generally, HIV-infected individuals experience a reduced level of high-density lipoprotein (HDL) cholesterol and low-density lipoprotein (LDL) cholesterol, and subsequently by a raised in plasma triglyceride (TG) in the years before they develop AIDS^14,15^. Even though it is known that prolong HAART use can result in dyslipidaemia, regular monitoring of lipids profile in HIV infected patients on HAART is not a conventional practice in Ghana, thus exposing infected individuals to a potentially high-risk metabolic and cardiovascular complications^15^. Abnormal metabolism of lipids leads to dyslipidaemia and others to atherosclerotic events that clinically manifest as coronary heart disease, myocardial infarction, and stroke^16,17^.

The metabolism of lipids is mediated by the enzymes, lecithin cholesterol acyltransferase (LCAT), and lipoprotein lipase (*LPL*). *LCAT* mediates the maturation of high-density lipoprotein (HDL) and reverses the transportation of cholesterol while *LPL* cleaves fatty acid and monoacylglycerol from triglycerides metabolism^18^. Alterations in the structure and function of these enzymes are also reported to affect the metabolism of lipids and lead to dyslipidaemia^19,20^. While abnormal lipid accumulation can be caused by both familial and secondary factors, little attention has been given to familial or functional cause of dyslipidaemia induced by HAART among HIV/AIDs infected individual in Ghana One way to assess the familial or genetic variations of the individual population is by employing the concept of single nucleotide polymorphism (SNPs).

SNPs have been recognised as one of the most prominent causes of mutations in these enzymes which predispose carriers to the development of dyslipidaemia and the resultant negative metabolic sequelae^19^. Previous prospective studies^14,21^ that found associations between *apo A-V* polymorphism and the development of dyslipidaemia in HIV infected individuals on HAART suggested that polymorphism genotyping could help identify prospective HAART patients who are at risk of developing dyslipidaemia^22,23^.

Recently, a study by Obirikorang et al. demonstrated the presence of SNPs in four candidate genes (ABCA1-rs2066714, LDLR-rs6511720, APOA5-rs662799, and DSCAML1-rs10892151) among HIV-infected individual on ART in a Ghanaian population^24^. The authors found an association between variants in APOA5-rs662799 polymorphisms and dyslipidaemia^24^. These studies provide evidence that long-term use of ART coupled with SNPs potentiate the development of dyslipidaemia among HIV infected individual. To minimize the effect of duration on HAART for dyslipidaemia and to explore the involvement of SNPs in dyslipidaemia among HIV infected individuals, we sequenced a total of 256 selected PCR amplicons, comprising 137 LPL (exons 3, 5 and 6) and 119 LCAT (exons 1, 4 and 6) among HIV-infected patients on HAART who developed dyslipidaemia within the two year period in a Ghanaian population.

## Methods

### Study design and population

This hospital-based case–control study among HIV-infected patients was undertaken from October 2016 to December 2019 at three-selected hospitals in the Northern region [Tamale West Hospital (TWH), Tamale Central Hospital (TCH) and the Tamale Teaching Hospital (TTH). The Committee for Human Publication and Research Ethics of the Kwame Nkrumah University of Science and Technology, Kumasi Ghana (ref/CHRPE/AP/367/16) and the Institutional Review Committee of the Ghana Health Service in the Northern region (ref/GHS/NR/18-0) approved the study protocol. Written informed consent in a form of signature or thumbprint was obtained from all participants, and confidentiality was assured. The study was conducted under the conditions of the declaration of Helsinki for human research.

### Sample size and selection of participants

Using an effect size of 0.31 in reference to finding from previous study^24^, and given an alpha (α) value of 0.05, 95% confidence interval, a power of detecting a statistically significant difference between two groups, based on the goodness-of fit tests (X^2^-test), the required total calculated sample size was 206. A cohort of 300 HIV-infected individuals was initially recruited using the medical records available at the Anti-Retroviral Treatment (ART) Centre.

Age, gender, date of the first diagnosis, weight, blood pressure measurement, and type of HAART were retrieved from medical records available at the Anti-Retroviral Treatment (ART) Centre using a well-structured, self-designed questionnaire. These questionnaires were pre-tested among 10 persons living with HIV administered and on HAART to clear possible ambiguity and difficulty in answering the questions.

### Inclusion and exclusion criteria

**Figure 1:**
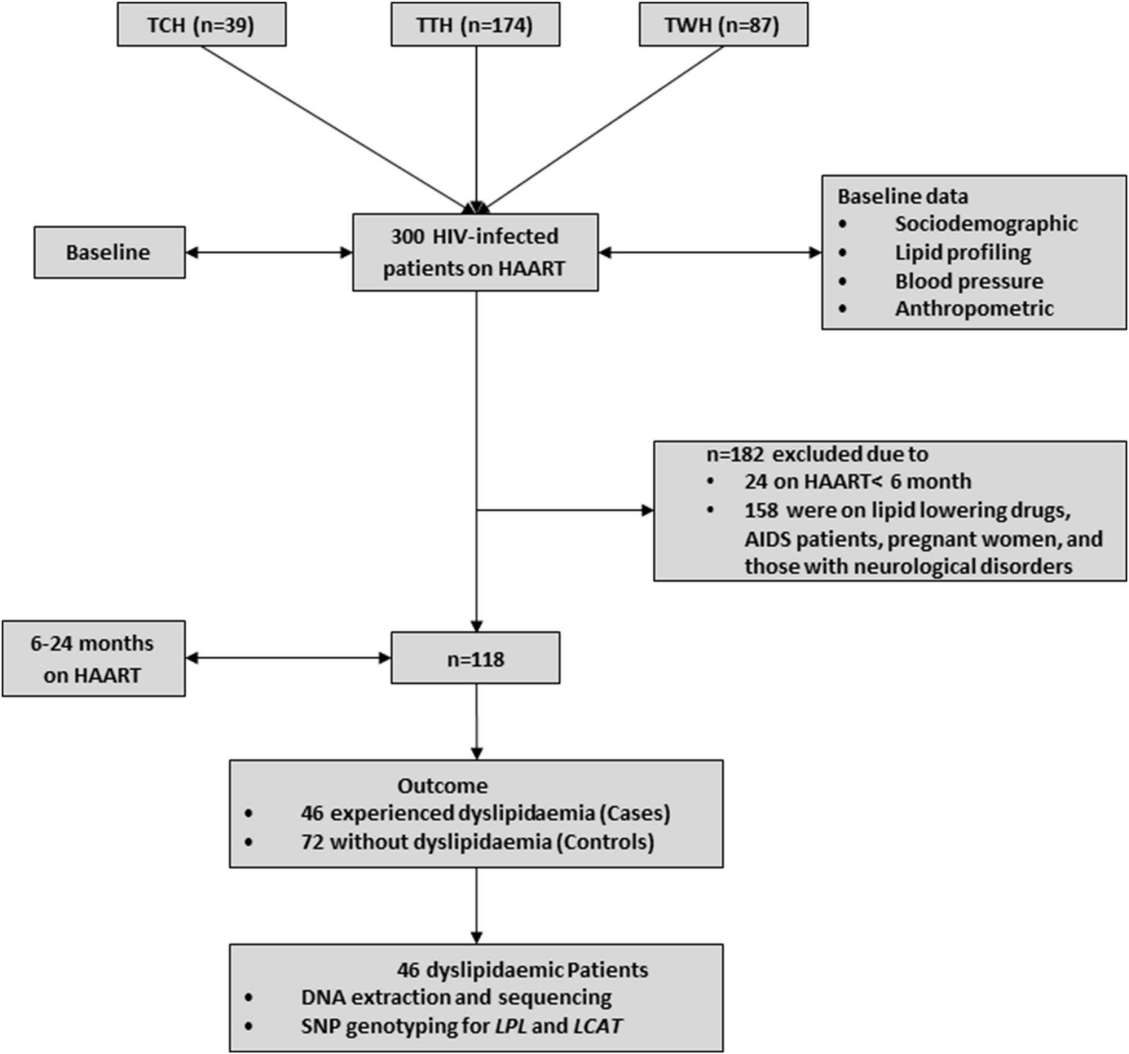
Flowchart of study participants. *TCH* Tamale Central Hospital, *TTH* Tamale Teaching Hospital, *TWH* Tamale West Hospital.

Long-term use of ART has been demonstrated to exponentiate the development of dyslipidaemia among HIV infected individual. To investigate the role of SNPs in dyslipidaemia among HIV infected individuals while minimizing the effect of duration on HAART for dyslipidaemia, we included individuals who has been on HAART for at most two years. A total of 118 HIV-infected individuals who had been on HAART for 6–24 months were included in the final analysis for this study (Fig. 1). The antiretrovirals included Zidovudine (AZT), Lamivudine (3TC), efavirenz (EFV), Nevirapine (NVP), Tenofovir (TDF), Lopinavir (LPV), NVP, Emtricitabine (FTC) and Ritonavir (r). These drugs were administered as triple combinations as follows; AZT/3TC/EFV, AZT/3TC/NVP, TDF/3TC/EFV, TDF/3TC/LPV, TDF/3TC/LPV/r, TDF/3TC/NVP, TDF/FTC/EFV.

Conversely, of the 300 HIV-infected individuals recruited at baseline, 182 patients comprising 24 patients who had been of HAART for less than 6 months and 158 who were on lipid lowering drugs, had neurological disorder, were pregnant women, and had AIDS were excluded from the study.

### Biochemical analysis

Venous blood sample (5 mL) was taken from these participants, 4 mL was dispensed into a gel vacutainer tube for the estimation of serum lipids using a fully automatic chemistry analyzer (Vital Scientific Selectra Flexor XL, UK) while 1 mL was dispensed into EDTA vacutainer tube for DNA extraction for PCR analysis. The Friedewald’s formula, LDL-C = TC-(HDL-C-TG/2.2) was used for calculating LDL-C. Friedewald’s equation over miscalculates LDL-c when serum triglycerides (TG) are high, therefore subjects with TG ≥ 4.2 mmol/L were excluded from the study. This is to avoid potential bias affecting the association of LDL-C with dyslipidaemia.

Dyslipidaemia was defined in this study as the presence of at least two lipid abnormalities (i) hypertriglyceridaemia—fasting serum triglyceride level ≥ 1.7 mmol/L (150 mg/dL), (ii) Reduced high density lipoprotein (HDL)-cholesterol, (iii) serum HDL-cholesterol ≤ 1.03 mmol/L (40 mg/dL) (males) or 1.29 mmol/L (≤ 50 mg/dL) (females), (iv) high serum low density lipoprotein (LDL)-cholesterol ≥ 3.37 mmol/L (130 mg/dL) and (v) high total cholesterol ≥ 6.2 mmol/L (200 mg/dL) as defined by NCEP ATP III^25^. Of the 118 participants, 46 HIV-infected patients had dyslipidaemia and were classified as cases whereas 72 of them had no dyslipidaemia and were classified as controls (Fig. 1).

### Selection of SNPs

Long-term usage of HAART is significantly associated with abnormal metabolism. To minimize the effect of the duration on HAART for dyslipidaemia and explore the role that SNPs play in dyslipidaemia among HIV infected individuals, two candidate SNPs (*LCAT* and *LPL*) were genotyped and sequenced among 46 HIV-infected patients who developed dyslipidaemia within the two years on being on HAART. These SNPs were selected based on reports from previous studies that demonstrate a significant association with dyslipidaemia following a review of PubMed reports and GWAS. Lecithin cholesterol acyltransferase (LCAT) mediates the maturation of high-density lipoprotein (HDL) and reverses the transportation of cholesterol while lipoprotein lipase (*LPL*) cleaves fatty acid and monoacylglycerol from triglycerides^18^.

### PCR conditions

All PCR reactions were performed using Mtaq polymerase, ddNTPs, Mg_2_Cl_3_, Nuclease-free sterile distilled water, genomic DNA, forward primer, reverse primer, and buffer. A total reaction volume of 40 µL comprising 2 µL Taq polymerase (1 in 25 dilutions), 6 µl PCR buffer, 6 µL MgCl, 2 µL gDNA, 2 µL each of primer pairs at 10 µM, and 17 µL of Nuclease-free sterile distilled water was used. PCR was run using the following cycling conditions: an initial denaturation of 95 °C for 10 min; 35 cycles of 95 °C for 1 min, (*Tm*^*a1*^5°C) for 1 min, and 68 °C for 2 min; a final extension of 68 °C for 10 min; and a holding temperature of 4 °C until the end of a run. All PCR runs were performed with GeneAmp PCR System 2700 following manufacturer’s protocols (Applied Biosystems, 850 Lincoln Centre Drive, Foster City, California 94404, USA) (Table 1).

**Table 1:** Primer information.

Primer name	Primer sequence	Amplicon size	TM (℃)
LCAT exon 1	FP: 5′-GCTTTCTCTGGCAGTAGGCA-3′ RP: 5′-TGGTGGGGGCTTACCGAG-3′	164	60.04 61.01
LCAT exon 4	FP: 5′-GTGCTGCTGGTCCCCC-3′ RP: 5′-TCATCCGCAGAGACACTCA-3′	95	59.61 58.04
LCAT exon 6	FP: 5′-TGTCCCACCTTGCTCCATATC-3′ RP: 5′-TCAACCTGAAACATAGCCATCA-3′	594	59.51 57.70
LPL exon 3	FP: 5′-AAAGCTTGTGTCATCATCTTCAGG-3′ RP: 5′-CACATAAGTCTCCTTCTCCCAGT-3′	180	59.54 59.23
LPL exon 5	FP: 5′-AATTTACAAATCTGTGTTCCTGCT-3′ RP: 5′-AGGATAAGAGTCACATTTAATTCGC-3′	234	57.34 57.52
LPL exon 6	FP: 5′-TGCCGAGATACAATCTTGGTGT-3′ RP: 5′-CCTTATTTACAACAGTCTCCAGCC-3′	243	59.76 59.36

The designed primers were synthesized by Integrated DNA Technologies, Coralville, IOWA, United States (https://eu.idtdna.com/site, integrated DNA Technologies Inc, USA).

*LCAT *lecithin cholesterol acyltransferase, *LPL* lipoprotein lipase, *FP* forward primer, *RP* reverse primer, *TM *melting temperature.

**Figure 2:**
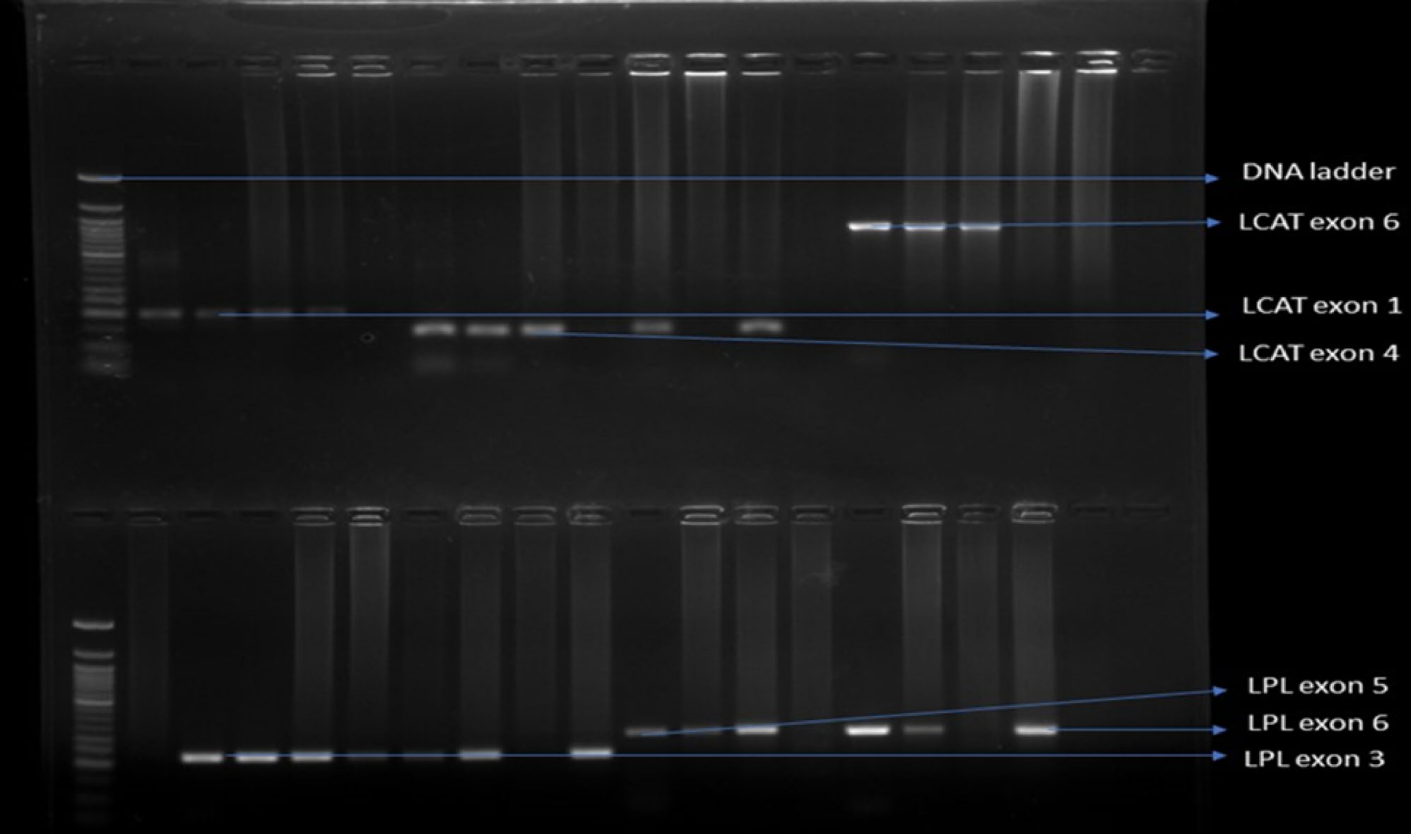
Results of ethidium bromide-stained full-length agarose gel electrophoresis visualized with UV light.

Figure 2 shows the various primer amplicons on agarose gel, along the DNA ladder (1000 bp). We designed primers that targeted exons 1, 4 and 6 in LCAT and exons 3, 5 and 6 in LPL, This full-length gel shows that, LCAT exon 6 is bigger than exon 1 and exon 1 is bigger than exon 4, since the smallest amplicon runs fasted (Fig. 2).

### DNA sequencing

A total of 256 selected PCR amplicons comprising 137 *LPL* (exons 3, 5 and 6) and 119 *LCAT* (exons 1, 4, and 6) samples were successfully sequenced at Inqaba Biotech, Pretoria, South Africa. DNA sequencing was done by the automated Sanger sequencing method^26^. The PCR amplified products were purified and sequenced under standard conditions on the ABI 3500XL Genetic Analyzer (POP7, Big Dye 3.1). The sequencing data was analyzed using Variant Reporter version 1.0 (Applied Biosystems, Foster City, CA) and Sequencher version 4.8 (Gene Codes Corporation, Ann Arbor, MI) according to Inqaba Biotech, Pretoria, South Africa. The sequence results were received and analysed using Chromas, Technelysium DNA sequencing software at https://technelysium.com.au/wp/chromas and multiple sequence alignment carried out using Clustalw at https://www.genome.jp/tools-bin/clustalw. Protein modelling was carried out using the ExPASy Bioinformatics Resource Portal at https://web.expasy.org/translate (Supplementary Figs. S1–S4).

### Statistical analysis

Data was entered into Microsoft excel 2016 and exported to GraphPad Prism version 6.0 (www.graphpad.com) for analysis. Data was presented as numbers, percentages, means, and 95% confidence interval and, median and interquartile range. The Kolmogorov–Smirnov test was used to check the normality of the continuous variables. Parametric continuous were compared between groups using student paired t-test and expressed as mean (95% CI) while non-parametric variables were performed with the Mann–Whitney test and expressed as median (inter-quartile range). Logistic regression models were fitted to test for associations between dyslipidaemia and selected single SNP. After the sequencing and alignment, some participants had DNA sequence same as the reference sequence and were classified as such. Other participants had DNA sequences different from the reference sequence and were classified as the SNP population. A P < 0.05 was considered statistically significant.

## Results

Table 2 shows a comparison between case and control subjects regarding demographic characteristics, weight measurement, haemodynamic and lipid parameters, and dyslipidaemic indices. In all, 39.0% (46/118) of the study population developed dyslipidaemia based on NCEP-ATPII definition and were classified as cases, while 61.0 (72/118) were without dyslipidaemia and were classified as controls. There were more females than males (99 vs. 19). The mean duration of ART drugs was 16 months. The case-subjects were significantly weightier than the control-subjects [65.5 vs. 59.3 p = 0.0071]. Higher levels of triglyceride (p = 0.0439), TC (p < 0.0001), LDL-C (p < 0.0001), non-HDL-C (p < 0.0001) and VLDL-C (p = 0.0449) were found in case-subjects than the control-subjects. Compared to the case-subjects, control-subjects recorded significantly higher levels of HDL-C [0.7 vs. 1.5, p < 0.0001].

**Table 2:** General characteristics of study participants.

Variable	Cases (n = 46)	Controls (n = 72)	P-value
Age (years)	42.3 (39.1–45.4)	37.5 (35.4–39.7)	0.0107
**Gender**^a^			0.9870
Male	7 (15.2)	12 (16.7)	
Female	39 (84.8)	60 (83.7)	
Duration of HAART (months)	15.6 (13.5–17.6)	15.7 (14.1–17.2)	0.9093
**HAART regimen**			
AZT/3TC/EFV	1 (2.2)	1 (1.4)	0.9989
AZT/3TC/NVP	7 (15.2)	6 (8.3)	0.3662
TDF/3TC/EFV	34 (73.9)	59 (82.0)	0.3577
TDF/3TC/NVP	4 (8.7)	6 (8.3)	0.9989
Systolic blood pressure	110.9 (106.5–115.2)	110.6 (107.2–114.1)	0.9353
Diastolic blood pressure	82.1 (79.4–84.9)	78.6 (76.8–80.4)	0.0300
Weight (kg)	65.5 (61.6–69.4)	60.0 (53.5–70.0)	0.0071
**Lipid parameters (mmol/L)**			
Total cholesterol	5.6 (5.3–5.8)	3.9 (3.6–4.2)	< 0.0001
Triglycerides	1.5 (1.3–1.8)	1.2 (1.0–1.4)	0.0439
HDL-cholesterol	0.7 (0.6–0.8)	1.5 (1.4–1.6)	< 0.0001
LDL-cholesterol	4.2 (4.0–4.4)	2.7 (2.5–2.9)	< 0.0001
VLDL-cholesterol	0.7 (0.6–0.8)	0.6 (0.5–0.7)	0.0449
Coronary risk	14.9 (11.8–17.9)	12.7 (10.2–15.2)	0.2782
Non-HDL cholesterol	4.9 (4.7–5.1)	3.2 (3.0–3.4)	< 0.0001
**Dyslipidaemic indices**			
High triglycerides, n (%)^a^	13 (28.3)	16 (22.2)	0.5138
High total cholesterol, n (%)^a^	46 (100.0)	10 (13.9)	< 0.0001
Low HDL-cholesterol, n (%)^a^	46 (100.0)	62 (86.1)	< 0.0001
High LDL-cholesterol, n (%)^a^	46 (100.0)	16 (22.2)	< 0.0001

Cases: HIV-infected patients with dyslipidemia, control: HIV-infected without dyslipidaemia, Data is presented mean (95% CI).

^a^Fisher exact test, Dyslipidaemia was defined as the presence of having at least two lipid abnormalities based on the NCEP-ATP III criteria. Zidovudine (AZT), Lamivudine (3TC), efavirenz (EFV), Nevirapine (NVP), Tenofovir (TDF), Lopinavir (LPV), NVP, Emtricitabine (FTC) and Ritonavir (r). Dyslipidaemic indices are presented as frequency (percentage).

**Figure 3:**
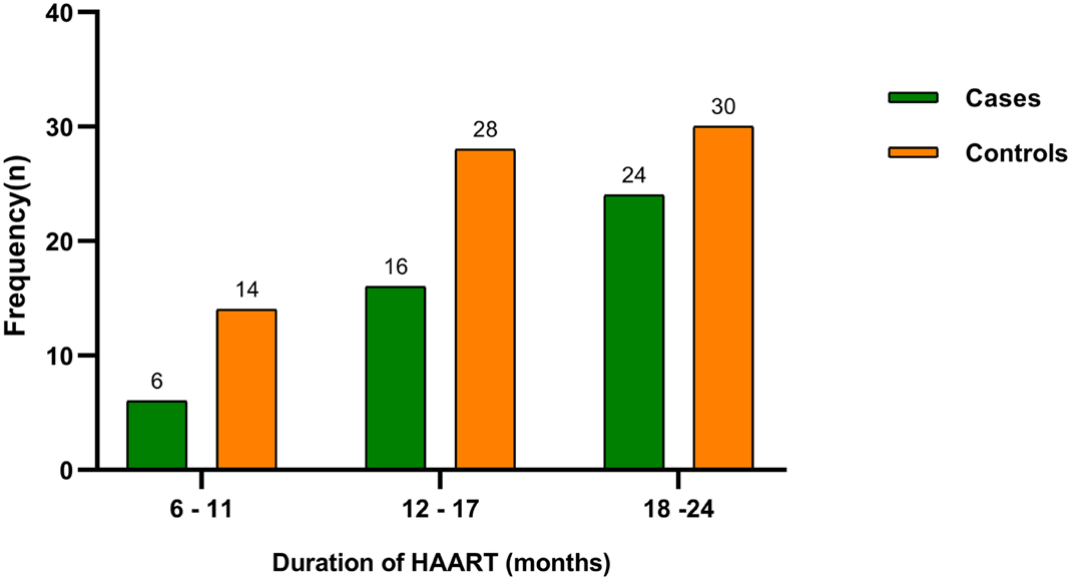
Distribution of the duration of HAART. Data are presented in frequency (n).

Figure 3 shows the distribution duration of HAART. The majority of the subjects had been on HAART between 18–24 months (45.8%) followed by 12–17 months (37.3%) and the least being 6–11 months (16.9%) respectively (Fig. 3).

### Single nucleotide polymorphism identified in LCAT

The study identified 5 substitutional SNPs in *LCAT* exon 1. At position 86 of *LCAT* exon 1, A was substituted for C in 12.5% (5/40) of the sample population while 87.5% (35/40) retained a C in the same position. This C → A nucleotide change did not significantly influence dyslipidaemia in the study population (p = 0.091). At position 89 of *LCAT* exon 1, A is substituted for G in 12.5% (5/40) of the sampled population while 87.5% (35/40) of the sampled population retained the G. This G → A SNP was not significantly associated with dyslipidaemia outcomes in HIV-infected individuals on HAART (p = 0.342). At position 97 of *LCAT* exon 1, there was a deletion of A in 32.5% (13/40) of the sampled population while 67.5% (27/40) of the sample population retained the A. This SNP was significantly associated with dyslipidaemic outcomes in the study population (p = 0.0004). At position 111, A was substituted for C in 7.5% (3/40) of the sampled population, while 92.5% (37/40) of the population retained C. This C → A nucleotide change was not significantly associated with dyslipidaemia in the study population (p = 0.231). Similarly, at position 121, 12.5% (5/40) of the study population expressed A while 87.5% (35/40) of the study population expressed C, this SNP was not significantly associated with dyslipidaemia in the study population (p = 0.342). Another SNP, a deletion of A was identified at position 8 of *LCAT* exon 4 in 91.3% (42/46) of the sampled population while 8.7% (4/46) of the study population retained an A at the same position. The deletion of A at position 8 of *LCAT* exon 4 was not significantly associated with dyslipidaemia outcomes in the sampled population (p = 0.990). No SNP was identified in LCAT exon 6 (Table 3).

**Table 3:** Types of nucleotide changes identified in LCAT, and their associations with dyslipidemia in the study population.

Mutation ∆	Exon	Position	Ref Seq (frequency)	SNP Seq (frequency)	OR (95% CI)	P-value
Substitution	1	86	C (33/40, 87.5%)	A (7/40, 12.5%)	8.143 (0.898–97.551)	0.091
Substitution		89	G (35/40, 87.5%)	A (5/40, 12.5%)	0.211 (0.021–2.079)	0.341
Deletion		97	A (27/40, 67.5%)	del (13/40, 32.5%)	28.500 (3.155–57.400)	0.0004
Substitution	4	111	C (37/40, 92.5%)	A (3/40, 7.5%)	8.200 (0.396–16.990)	0.231
Substitution		121	C (35/40, 87.5%)	A (5/40, 12.5%)	4.750 (0.481–46.910)	0.341
Deletion		8	A (4/46, 8.7%)	del (42/46, 91.3%)	0.500 (0.041–6.086)	0.999

Odds ratio for dyslipidaemia risk of the SNPs identified in *LCAT* exon 1 and 4. *REF *reference, *SEQ* sequence, *OR *odds ratio, *CI* confidence interval, *del* deletion, ∆ change, Data are presented as discrete, fractions and percentages. P-values < 0.05 considered statistically significant. Reference population were defined as individuals with ‘normal’ nucleotide sequenced whereas SNP population were those with nucleotide changes.

Table 4 shows the types of nucleotide changes identified in *LPL* and their associations with dyslipidaemia in the study population**.** A total of 25 SNPs were identified in exons 5 and 6 of *LPL*; 22 were substitutions, and 3 were insertions. At positions 1, 2 and 3 of *LPL* exon 3, 8.7% (4/46) of the sampled population had TTA while 91.3% (42/46) of the study population had GCC. This SNP was not significantly associated with dyslipidaemia in the study population (p = 0.279). At positions 5 and 6, 8.7% (4/46) of the study population had AA while 91.3% (42/46) of the study population had GC. These GC → AA nucleotide changes at positions 5 and 6 of *LPL* exon 5 were not significantly associated with dyslipidemia in the population sampled (p = 0.279).

**Table 4:** Types of nucleotide changes identified in *LPL*, and their associations with dyslipidaemia in the study population.

Mutation ∆	Exon	Position	Ref seq frequency	SNP seq frequency	OR (95% CI)	P-Value
Insertion	5	8	(39/46, 84.8%)	C (3/46, 6.5%)	3.86 (0.17–87.20)	0.502
				T (4/46, 8.7%)	5.40 (0.26–11.37)	0.261
		27, 28, 29	(42/46, 91.3%)	TAC (4/46, 8.7%)	4.85 (0.23–10.17)	0.279
Substitution	6	1, 2, 3	GCC (42/46, 91.3%)	TTA (4/46, 8.7%)	4.85 (0.23–10.17)	0.279
		5, 6	CG (42/46, 91.3%)	AA (4/46, 8.7%)	4.85 (0.23–10.17)	0.279
		9,10,11,12	GTCG (43/46, 94.3%)	CATG (3/46, 6.5%)	3.29 (0.15–74.06)	0.519
		14	C (42/46, 91.4%)	T (4/46, 8.7%)	4.85 (0.23–10.17)	0.279
		15	T (43/46, 94.3%)	C (3/46, 6.5%)	3.29 (0.15–74.06)	0.519
		17	T (39/46, 85.8%)	G (3/46, 6.5%)	3.86 (0.17–87.20)	0.502
				C (4/46, 8.7%)	5.40 (0.26–113.7)	0.26
		20	C (39/46, 85.8%)	A (3/46, 6.5%)	3.86 (0.17–87.20)	0.502
				G (4/46, 8.5%)	5.40 (0.26–113.7)	0.260
		21	C (43/46, 94.3%)	A (3/46, 6.5%)	3.29 (0.14–74.06)	0.519
		23	G (42/46, 91.5%)	A (4/46, 8.7%)	4.85 (0.23–101.7)	0.279
		33	C (42/46, 91.5%)	T (4/46, 8.7%)	4.85 (0.23–101.7)	0.279
		39	T (43/46, 94.3%)	G (3/46, 6.5%)	3.29 (0.15–74.11)	0.519
		41, 42, 43, 44, 45	GTAGA (40/46, 87.0%)	GTATG (3/46, 6.5%)	0.11 (0.005–2.55)	0.148
				ACCCG (3/46, 6.5%)	0.11 (0.005–2.55)	0.148
		47, 48, 49	GTC (40/46, 87.0%)	TAA (3/46, 6.5%)	2.56 (0.11–58.40)	0.990
				GAC (3/46, 6.5%)	0.10 (0.004–2.34)	0.133
		16	T (40/43, 93.0%)	G (3/43, 7.0%)	0.103 (0.005–2.14)	0.089
		80	A (41/43, 95.3%)	T (2/43, 4.7%)	5.00 (0.22–11.50)	0.489
		86	C (35/43, 81.6%)	T (8/43, 18.6%)	1.18 (0.23–6.13)	0.990
		96	T (39/43, 90.7%)	A (4/43, 9.3%)	9.51 (0.47–19.01	0.110
		190	C (41/43, 95.3%)	T (2/43, 5.1%)	4.74 (0.21–105.6)	0.489

Odds ratio for dyslipidaemia risk of the various SNPs identified in *LPL*. *SEQ* sequence, *FREQ* Frequency, *REF* Reference, ∆ change, *OR* Odds ratio, *LPL* lipoprotein lipase. Data are presented as discrete, fractions and percentages. P-values < 0.05 considered statistically significant. Reference population were defined as individuals with ‘normal’ nucleotide sequenced Whereas SNP population were those with nucleotide changes. SNPs identified in LCAT and their contributions to dyslipidaemia in the study population.

At position 8 of *LPL* exon 5, 6.5% (3/46) of the sampled population had C inserted into their genomes, 8.7% (4/46) of the population had T inserted at the same position, while 85.8% (39/46) had neither C nor T inserted into their genomes. The insertion of C and T at position 8 of *LPL* exon 5 did not have any significant effect on dyslipidaemic outcomes in HIV infected individuals on HAART. No SNP identified in *LPL* exon 3 (Table 4).

### SNPs identified in *LCAT* and their contributions to dyslipidaemia in the study population

A total of 5 SNPs were identified in LCAT exon 1 (at positions 86, 89, 97, 111) while 1 SNP was identified in *LCAT* exon 6, at position 121. At position 86 of *LCAT* exon 1, 12.5% of the samples collected have A in place of C present in the reference population. There was no statistically significant difference between the SNP population and the reference population in relation plasma TC (p = 0.070), TG (p = 0.530), LDL-C (p = 0.490), VLDL-C (p = 0.230) and CR (p = 0.156), respectively. The plasma HDL concentration was significantly higher in the SNP population compared with in the reference population (1.07 ± 0.25 mmol/L vs. 0.881 ± 0.523 mmol/L, p = 0.0004). At position 89 of *LCAT* exon 1, 12.5% of the sample population has A in place of G in 87.2% of the study population. There was no statistically significant difference between the SNP and the reference population for the lipid parameters (p > 0.05) (Table 5).

**Table 5:** Comparison of lipid profile results amongst reference sequence and SNP populations.

Nucleotide ∆	Exon	POS	Reference population			SNPs population		
			SEQ FREQ	CHOL	TRIG	SEQ FREQ	CHOL	TRIG
Substitution	1	86	C*(35/40, 87.5%)	6.483 ± 1.512	1.337 ± 0.873	A (5/40, 12.5%)	4.538 ± 1.241	1.966 ± 1.609
		89	G* (35/40, 87.5%)	4.982 ± 1.521	0.570 ± 0.255	A (5/40, 12.5%)	4.156 ± 0.887	1.572 ± 1.051
		97	A*(27/40, 67.5%)	4.208 ± 1.119	1.339 ± 0.952	*del* (13/40, 32.5%)	6.272 ± 1.114^†^	1.671 ± 1.213
		111	C*(37/40, 92.5%)	4.684 ± 1.344	1.328 ± 0.838	A (3/40, 7.5%)	7.283 ± 0.719	2.917 ± 2.228
		121	C*(35/40, 87.5%)	4.688 ± 1.368	1.337 ± 0.850	A (5/40, 12.5%)	6.214 ± 1.660^††^	2.220 ± 1.886
Deletion	4	8	A*(4/46, 8.7%)	6.177 ± 2.154	0.727 ± 0.273	*del* (42/46, 91.3%)	4.842 ± 1.484	1.564 ± 1.005

			SEQ FREQ	HDL	LDL	SEQ FREQ	HDL	LDL
Substitution	1	86	C*(35/40, 87.5%)	1.071 ± 0.246^†^	3.331 ± 1.320	A (5/40, 12.5%)	0.881 ± 0.523	5.017 ± 1.407
		89	G*(35/40, 87.5%)	0.969 ± 0.477	3.699 ± 1.546	A (5/40, 12.5%)	0.530 ± 0.430	3.120 ± 0.578
		97	A*(27/40, 67.5%)	0.979 ± 0.408^††^	2.985 ± 1.176	*del* (13/40, 32.5%)	883 ± 0.5280	4.958 ± 1.079^††^
		111	C*(37/40, 92.5%)	0.906 ± 0.506	3.459 ± 1.393	A (3/40, 7.5%)	1.013 ± 0.207	5.687 ± 0.458
		121	C*(35/40, 87.5%)	0.902 ± 0.520	3.463 ± 1.421	A (5/40, 12.5%)	0.998 ± 0.155	4.772 ± 1.409
Deletion	4	8	A*(4/46, 8.7%)	1.393 ± 0.534	4.637 ± 2.195	*del* (42/46, 91.3%)	0.966 ± 0.513	3.563 ± 1.555

Unpaired t-test comparison of lipid profile parameters. ^†^Denotes a significant comparison between reference population and SNP population when P-value is < 0.05, ++-^† ††^Denotes a significant comparison between reference population and SNP population when P-value is < 0.001. *SEQ *sequence, *LDL* low density lipoprotein, *HDL *high density lipoprotein, *TRIG *triglycerides, *VLDL* very low-density lipoprotein, *CR* coronary risk. *FREQ *frequency, ∆ change, *LCAT *lecithin cholesteryl acyl transferase, Data are presented as means ± SD, discrete, fractions and percentages. Reference population were defined as individuals with ‘normal’ nucleotide sequenced Whereas SNP population were those with nucleotide changes.

## Discussion

HAART administration interferes with triglyceride-rich lipoprotein hydrolysis by interfering with its binding to lipoprotein lipase, thereby hindering normal chylomicron, LDL, VLDL catabolism, trapping of fatty acids in peripheral adipose tissues and use by muscles^27^. HAART also interferes with the degradation of the nuclear transcriptionally active factor SREBP1 (nSREBP1), which is the master transcription control protein involved in plasma lipid synthesis^27,28^. The nSREBP1, therefore, lingers in the nucleus and continuously stimulate the transcription and translation of genes involved in the lipid biosynthesis pathway^29,30^.

The prevalence of dyslipidaemia observed in the present study was 39.0%, which is consistent with cross-sectional studies and case–control studies in Ghana and other populations^24,31–33^. In the present study, dyslipidaemia was defined by increased plasma total cholesterol, LDL-C and decreased HDL concentrations, which is similar to the criteria used in cross-sectional studies by Hu et al.^34^, and Chattopadhyay and Aldous^35^. The latter studies demonstrated that hypercholesterolaemia, hypertriglyceridaemia, and hypoalphalipoproteinaemia together with lipodystrophy were principal indices for dyslipidaemia among HIV-infected individuals after HAART exposure^35^.

Genetic factors have been shown to contribute to dyslipidaemia in HIV-infected individuals on HAART. The present study identified five (5) SNPs (at position 86, 89, 97, 111 and 121) in *LCAT* exon 1 and one (1) SNP (at position 8) in *LCAT* exon 4. Out of the 6 SNPs identified in LCAT, only 1 SNP was clinically significant; a deletion of adenine (A) at position 97 of *LCAT* exon 1 in 32.5% of the sampled population. This study observed significant dyslipidaemia in participants with this SNP at position 97 of *LCAT* exon 1. The presence of this SNP results in a frameshift in the nucleotide sequence of the *LCAT* mRNA produced and hence a change in the amino acid sequence of the polypeptide produced (Supplementary Figs. S2, S3, and S4).

Protein modelling at ExPASy bioinformatic resource portal revealed that the deletion of adenine (A) at position 97 of *LCAT* exon 1 leads to a shift in the open reading frame, resulting in the replacement of asparagine (N) with histidine (H) at position 5 of the mutant *LCAT* (Supplementary Figs. S2, S3, and S4, https://www.expasy.org/proteomics/protein_structure). This results in a change in the first 5 N-terminal amino acid sequences of the mature mutant *LCAT*. The 4 to 8 N-terminal amino acids located in the membrane-binding domain of the normal *LCAT* enzyme play a critical role in LCAT’s recognition, specificity, selectivity, and binding to apolipoprotein A-I (ApoA-I) in HDL particles^36,37^.

A change in the first 5 N-terminal amino acids, therefore, impairs the function of the membrane-binding domain of the mutant LCAT’s, affecting its ability to specifically recognize, select and bind to its substrate (HDL). This change in the type and sequence of the first five amino acids in the mutant LCAT’s structure severely impairs activation and catalytic activity of the mutant enzyme^38^ accounting for the reduction in the rate of cholesterol esterification and reverse transport to the liver for clearance from the body. The cholesterol therefore accumulated in the body, leading to dyslipidaemia observed among the present study participants with this SNP. This is consistent with the findings of Francone and Fielding^39^ who documented that, *LCAT* has amino (N) and carboxyl (C) terminal extensions that are not predicted to have significant secondary structure. However, the *LCAT* N-terminus amino acid residues 2 to 5 of the mature protein is known to be important for LCAT activity, mediating contacts with apolipoprotein A-I (ApoA-I) in HDL particles. The amino acid residues 2–5 represent the macromolecular interaction site for HDL particles^39^. Murray et al.^40^ and Vanloo et al.^41^ documented in separate site-directed mutagenesis and antibody-binding experiments that, amino acid residues in the N-terminal region of *LCAT* play a structural role and ApoA-1’s binding to this site leads to the activation of LCAT.

This study’s findings also agree with the findings of Manthei et al.^37^ and Glukhova et al.^36^ who also documented that truncations in the N-terminus are critical for *LCAT* activity on HDL as it contains residues that are critical components of the HDL membrane-binding domain. Manthei et al.^37^ and Glukhova et al.^36^ also concluded that amino acid residues 4–8 have backbone amines that are critical for mediating interaction with ApoA-1 on HDL particles, substrate recognition, specificity, and selectivity.

Schindler et al.^42^ and Dube et al.^43^ also reported that *LCAT* glycoprotein has 4 N-glycosylation (Asn 20, 84, 272, and 384) and 2 sites of O-glycosylation sites (Thr407 and Ser409). The carbohydrate component constitutes 25% of LCAT’s total mass, the majority being N-linked^44–46^. Neuraminidase removal of the carbohydrate moiety of human *LCAT* led to a 60% decrease in the activity of the enzyme^42,45–47^. Their finding supports the finding of this study that a change in a functional domain of *LCAT* affects the activity of the enzyme. The change in the first 5 amino acids decreases the activity of the mutant LCAT without affecting *LCAT* protein synthesis and secretion^42,44,48^.

The influence of this SNP identified at position 97 of the mutant *LCAT* on plasma HDL cholesterol concentration is evidenced by the findings that hypoalphalipoproteinemia is one of the highest prevalent form of dyslipidemia present in this study. This shows the significant clinical influence of this SNP in inducing dyslipidaemia in HIV-infected individuals on HAART. In this study, 5 other SNPs (4 in LCAT exons 1 and 1 in exon 4) identified in *LCAT* do not have any significant effect on the dyslipidaemic outcomes in the study population. Twenty-five (25) SNPs were identified in *LPL* but none of these SNPs had a significant effect on the dyslipidaemic outcomes in the study population. This explains the observation that plasma triglycerides and VLDL concentrations did not change significantly after HAART.

Despite these findings, there were some limitations such as the sample size of the study was small, and SNPs present in other enzymes involved in the metabolism of lipids in the body were not sequenced. Also, the study did not longitudinally assess lipid parameters for the HIV-infected individuals which would have substantiated the association of SNPs presence and dyslipidaemia at baseline. Notwithstanding our observed findings are in line with previous reports.

## Conclusion

The study has demonstrated the existence of SNPs in LCAT and LPL among HIV-infected individuals with dyslipidaemia in Ghana. The presence of SNPs at position 97 of *LCAT* exon 1 leads to the formation of a mutant *LCAT* which potentially reduces the rate of cholesterol esterification and reverse transportation to the liver for excretion. This supports the observation that reduced HDL-C was one of the commonest types of lipid abnormalities among the study population. The SNPs identified in *LPL* did not significantly affect TG levels, supporting the observation that total triglycerides and VLDL concentrations did not change significantly after HAART. This study recommends the use of genotyping to prospectively identify individuals with SNPs that contribute to dyslipidaemia. Further longitudinal studies are required in larger HIV cohort to ascertain the findings in the present study.

## Supplementary information

Supplementary Information.

## Data Availability

The datasets used and/or analysed during the current study are available from the corresponding author on reasonable request.
