# Supplementary material for: Single nucleotide polymorphisms in LCAT may contribute to dyslipidaemia in HIV-infected individuals on HAART in a Ghanaian population
Source: Sci Rep. 2020 Nov 10;10:19419. doi: 10.1038/s41598-020-76113-2 (PMC7655843; doi:10.1038/s41598-020-76113-2)
Supplement: Supplementary file 1 — Supplementary Information. [file 41598_2020_76113_MOESM1_ESM.pdf]

Single nucleotide polymorphisms in LCAT may contribute to dyslipidaemia in HIV-infected individuals on HAART in a Ghanaian population

Simon Bannison Bani[1†], Kwabena Owusu Danquah[3†], Christian Obirikorang[2†*], William K.B.A. Owiredu[2], Lawrence Quaye[1], Edmund Dei[4], Emmanuel Acheampong [2, 5], Yussif Adams[1], Peter Paul M. Dapare[1], Moses Banyeh[1], Enoch Odame Anto[3, 5], Samuel Asamoah Sakyi [2]

†Equal contribution

[1]Department of Biomedical Laboratory Science, School of Allied Health Sciences, University for Development studies, Tamale, Ghana

[2]Department of Molecular Medicine, School of Medicine and Dentistry, College of Health Sciences, Kwame Nkrumah University of Science and Technology, Kumasi, Ghana

[3]Department of Medical Diagnostics, Faculty of Allied Health Sciences, College of Health Sciences, Kwame Nkrumah University of Science and Technology, Kumasi, Ghana

[4]Department of Pathology, School of Medicine and Health Sciences, University for Development studies, Tamale, Ghana

[5] School of Medical and Health Sciences. Edith Cowan University, Joondalup, Australia

Supplementary figures

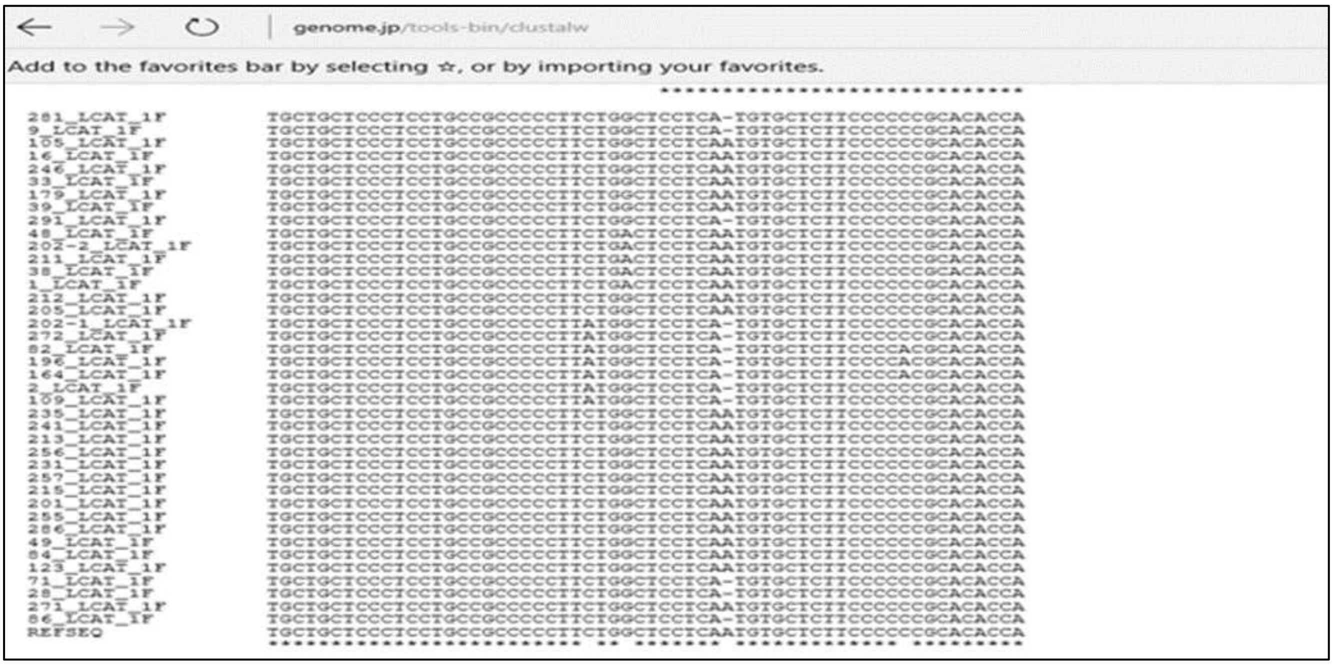

Figure S1. Clustalw alignment, showing (-) sites of Adenine (A) deletion in mutant LCAT exon 1

Figure S1 shows Clustalw alignment, showing (-) sites of Adenine (A) deletion in mutant LCAT exon 1.

NORMAL POPULATION	SNP POPULATION
**LCAT EXON 1 NORMAL NUCLEOTIDE SEQUENCE**<br>CCAGGGCTGGAATGGGGCCGCCCGGCTCCCCATGGCAGTGGGTG<br>ACGCTGCTGCTGGGGCTGCTGCTCCCTCCTGCCGCCCCCTTCTG<br>GCTCCTCAATGTGCTCTTCCCCCCGCACACCACGCCCAAGGCTG<br>AGCTCAGTAACCACACACGGCCCGTCATCCTCG	**LCAT (WITH SNP) EXON 1 NUCLEOTIDE SEQUEN**<br>CCAGGGCTGGAATGGGGCCGCCCGGCTCCCCATGGCAGTG<br>ACGCTGCTGCTGGGGCTGCTGCTCCCTCCTGCCGCCCCCT<br>GCTCCTCA-TGTGCTCTTCCCCCCGCACACCACGCCCAAG<br>CTGAGCTCAGTAACCACACACGGCCCGTCATCCTCG
**AMINO ACID SEQUENCE**<br>**FWLLNV LFPPHTTPKA**<br>**ELSNHTRPVI L**VPGCLGNQL EAKLDKPDVV NWMCYRKTED<br>FFTIWLDLNM FLPLGVDCWI DNTRVVYNRS SGLVSNAPGV<br>QIRVPGFGKT YSVEYLDSSK LAGYLHTLVQ NLVNNGYVRD<br>ETVRAAPYDW RLEPGQQEEY YRKLAGLVEE MHAAYGKPVF<br>LIGHSLGCLH LLYFLLRQPQ AWKDRFIDGF ISLGAPWGGS<br>IKPMLVLASG DNQGIPIMSS IKLKEEQRIT TTSPWMFPSR<br>MAWPEDHVFI STPSFNYTGR DFQRFFADLH FEEGWYMWLQ<br>SRDLLAGLPA PGVEVYCLYG VGLPTPRTYI YDHGFPYTDP<br>VGVLYEDGDD TVATRSTELC GLWQGRQPQP VHLLPLHGIQ<br>HLNMVFSNLT LEHINAILLG AYRQGPPASP TASPEPPPPE	**AMINO ACID SEQUENCE**<br>**LMAPHV LFPPHTT**<br>**ELSNHTRPVI L**VPGCLGNQL EAKLDKPDVV NWMCYRK<br>FFTIWLDLNM FLPLGVDCWI DNTRVVYNRS SGLVSNA<br>QIRVPGFGKT YSVEYLDSSK LAGYLHTLVQ NLVNNGY<br>ETVRAAPYDW RLEPGQQEEY YRKLAGLVEE MHAAYGK<br>LIGHSLGCLH LLYFLLRQPQ AWKDRFIDGF ISLGAPW<br>IKPMLVLASG DNQGIPIMSS IKLKEEQRIT TTSPWMF<br>MAWPEDHVFI STPSFNYTGR DFQRFFADLH FEEGWYM<br>SRDLLAGLPA PGVEVYCLYG VGLPTPRTYI YDHGFPY<br>VGVLYEDGDD TVATRSTELC GLWQGRQPQP VHLLPLH<br>HLNMVFSNLT LEHINAILLG AYRQGPPASP TASPEPP

Figure S2. Nucleotide and amino acid sequences of normal and mutant (with SNP) LCAT

Figure S2 shows the Adenine (in blue) residue which is deleted in the mutant LCAT sequence. The four (4) sites of N-glycosylation are shown in red. The first 27 N-terminal amino acids coded for by exon 1 are shown in bold. The first 5 N-terminal amino acids had Histidine (H) substituted for asparagine (N) at the site of SNP in the mutant LCAT, leading to the replacement of FWLLN (normal LCAT) for (LMAPH) in the mutant LCAT (in green).

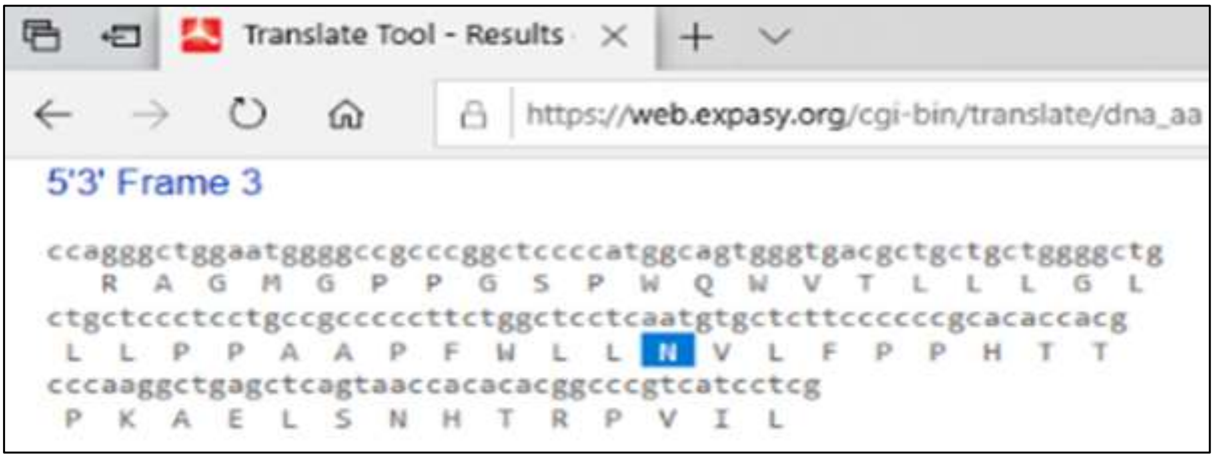

Figure S3. Result of normal LCAT exon 1 nucleotide sequence

Figure S3 shows expasy translate result of normal *LCAT* exon 1 nucleotide sequence, showing asparagine (N) in its normal position.

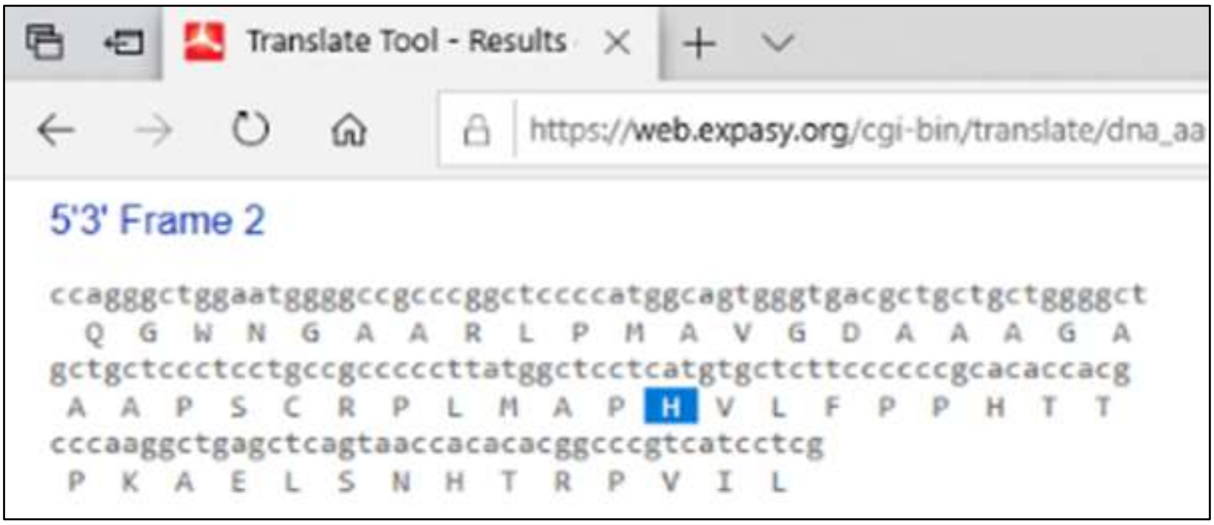

Figure S4. Result of mutant enzyme nucleotide sequence

Figure S4 depicts expasy translate result of mutant enzyme nucleotide sequence showing the Histidine (H) substituted for asparagine (N) at the SNP site.

Table 3: Association between identified nucleotide changes in LCAT and dyslipidemia in the study population

MUTATION ∆	EXON	POSITION	SNP	Dyslipidaemia (Yes)	Dyslipidaemia (No)	OR (95% CI)	P-Value
SUBSTITUTION		86	C	6(85.7%)	1(14.3%)	8.14(1.00-97.55)	0.090
			A	14(42.4%)	19(57.6%)	1	
SUBSTITUTION		89	A	1(16.7%)	4(83.3%)	0.21(0.02-2.08)	0.340
			G	19(54.3%)	16(45.7%)	1	
DELETION	1	97	-	12(92.3%)	1(7.7%)	28.5(3.16 - 57.4)	0.0004
			A	8(29.6%)	19(70.4%)	1	
SUBSTITUTION		111	C	3(100.0%)	0(0.0%)	8.2(0.39-16.90)	0.230
			A	17(45.9%)	20(54.1%)	1	
SUBSTITUTION		121	A	4(83.3%)	1(16.7%)	4.8(0.48-46.91)	0.340
			C	16(45.7%)	19(54.3%)	1	
DELETION	4	8	-	16(50.0%)	16(50.0%)	0.5(0.04-6.09)	0.990
			A	2(66.6%)	1(33.3%)	1	

Odds ratio for dyslipidaemia risk of the SNPs identified in *LCAT* exon 1 and 4. REF - reference, SEQ –sequence, OR-odds ratio, CI: Confidence Interval, del – deletion, ∆ - change, Data are presented as discrete, fractions and percentages. P-values < 0.05 considered statistically significant. The Woolf method was used when one (or more) value is zero, then 0.5 was added to each value before calculating the odds ratio and its confidence interval.

Table 3: Association between identified nucleotide changes in LCAT and dyslipidemia in the study population

MUTATION ∆	EXON	POSITION	SNPs	Dyslipidemia (Yes) n(%)	Dyslipidemia (No) n(%)	OR (95% CI)	P-Value
INSERTION	5	8	C	2(100.0%)	0(0.0%)	3.86(0.17-87.20)	0.50
			-	17(56.7%)	13(43.3%)	1	
			T	3(100.0%)	0(0.0%)	5.40(0.26-11.37)	0.26
			-	17(56.7%)	13(43.3%)	1	
		27, 28, 29	TAC	3(100.0%)	0(0.0%)	4.85(0.23-10.17)	0.28
			-	19(59.4%)	13(40.6%)	1	
SUBSTITUTION	5	1, 2, 3	TTA	3(100.0%)	0(0.0%)	4.85(0.23-10.17)	0.28
			GCC	19(59.4%)	13(40.6%)	1	
		5, 6	AA	3(100.0%)	0(0.0%)	4.85(0.23-10.17)	0.28
			GG	19(59.4%)	13(40.6%)	1	
		9,10,11,12	CATG	2(100.0%)	0(0.0%)	3.29(0.15-74.06)	0.52
			GTGG	20(60.6%)	13(39.4%)	1	
		14	C	3(100.0%)	0(0.0%)	4.85(0.23-10.17)	0.28
			T	19(59.4%)	13(40.6%)	1	
		15	T	2(100.0%)	0(0.0%)	3.29(0.15-74.06)	0.52
			C	20(60.6%)	13(39.4%)	1	
		17	C	2(100.0%)	0(0.0%)	3.86(0.17-87.20)	0.50
			T	17(56.7%)	13(43.3%)	1	
		20	A	2(100.0%)	0(0.0%)	3.86(0.17-87.20)	0.50
			C	17(56.7%)	13(43.3%)	1	
		21	A	2(100.0%)	0(0.0%)	3.29(0.14-74.06)	0.52
			C	20(60.6%)	13(39.4%)		
		23	A	3(100.0%)	0(0.0%)	4.85(0.23-101.7)	0.28
			G	19(59.4%)	13(40.6%)	1	
		33	T	3(100.0%)	0(0.0%)	4.85(0.23-101.7)	0.28
			C	19(59.4%)	13(40.6%)	1	
		39	G	2(100.0%)	0(0.0%)	3.29(0.15 -74.11)	0.52

		T	20(60.6%)	13(39.4%)	1	
	41, 42, 43, 44, 45	ACCCG	0(0.0%)	2(100.0%)	0.11(0.005-2.55)	0.15
		GTAGA	20(64.5%)	11(35.5%)	1	
		TAA	2(100.0%)	0(0.0%)	2.56(0.11-58.40)	0.99
	47, 48, 49	GTC	20(66.7%)	10(33.3%)	1	
		GAG	0(0.0%)	2(100.0%)	0.10(0.004-2.34)	0.13
		GTC	20(66.7%)	10(33.3%)	1	
6	16	G	0(0.0%)	3(100.0%)	0.10(0.005-2.14)	0.09
		T	20(57.1%)	15(42.9%)	1	
	80	T	2(100.0%)	0(0.0%)	5.00(0.22-11.50)	0.49
		A	18(50.0%)	18(50.0%)	1	
	86	T	4(57/1%)	3(42.9%)	1.18(0.23-6.13)	0.99
		C	17(53.1%)	15(46.9%)	1	
	96	A	4(100.0%)	0(0.0%)	9.51(0.47-19.01	0.11
		T	17(48.6%)	18(51.4%)	1	
	190	T	2(100.0%)	0(0.0%)	4.74(0.21-105.6)	0.49
		C	19(51.4%)	18(48.6%)	1	

Odds ratio for dyslipidaemia risk of the SNPs identified in *LCAT* exon 1 and 4. REF - reference, SEQ –sequence, OR-odds ratio, CI: Confidence Interval, del – deletion, **Δ** - change, Data are presented as discrete, fractions and percentages. P-values < 0.05 considered statistically significant, The Woolf method was used when one (or more) value is zero, then 0.5 was added to each value before calculating the odds ratio and its confidence interval.
